# Functional Genomic Analysis Identifies Indoxyl Sulfate as a Major, Poorly Dialyzable Uremic Toxin in End-Stage Renal Disease

**DOI:** 10.1371/journal.pone.0118703

**Published:** 2015-03-26

**Authors:** Sachin Jhawar, Prabhjot Singh, Daniel Torres, Francisco Ramirez-Valle, Hania Kassem, Trina Banerjee, Igor Dolgalev, Adriana Heguy, Jiri Zavadil, Jerome Lowenstein

**Affiliations:** 1 Department of Medicine New York University Langone Medical Center, New York, NY, United States of America; 2 Department of Dermatology, University of California San Francisco, San Francisco, CA, United States of America; 3 Department of Pathology and Center for Health Informatics and Bioinformatics, New York University Langone Medical Center, New York, NY, United States of America; Medical University of Graz, AUSTRIA

## Abstract

**Background:**

Chronic renal failure is characterized by progressive renal scarring and accelerated arteriosclerotic cardiovascular disease despite what is considered to be adequate hemodialysis or peritoneal dialysis. In rodents with reduced renal mass, renal scarring has been attributed to poorly filtered, small protein-bound molecules. The best studied of these is indoxyl sulfate (IS).

**Methods:**

We have attempted to establish whether there are uremic toxins that are not effectively removed by hemodialysis. We examined plasma from patients undergoing hemodialysis, employing global gene expression in normal human renal cortical cells incubated in pre- and post- dialysis plasma as a reporter system. Responses in cells incubated with pre- and post-dialysis uremic plasma (n = 10) were compared with responses elicited by plasma from control subjects (n = 5). The effects of adding IS to control plasma and of adding probenecid to uremic plasma were examined. Plasma concentrations of IS were measured by HPLC (high pressure liquid chromatography).

**Results:**

Gene expression in our reporter system revealed dysregulation of 1912 genes in cells incubated with pre-dialysis uremic plasma. In cells incubated in post-dialysis plasma, the expression of 537 of those genes returned to baseline but the majority of them (1375) remained dysregulated. IS concentration was markedly elevated in pre- and post-dialysis plasma. Addition of IS to control plasma simulated more than 80% of the effects of uremic plasma on gene expression; the addition of probenecid, an organic anion transport (OAT) inhibitor, to uremic plasma reversed the changes in gene expression.

**Conclusion:**

These findings provide evidence that hemodialysis fails to effectively clear one or more solutes that effect gene expression, in our reporter system, from the plasma of patients with uremia. The finding that gene dysregulation was simulated by the addition of IS to control plasma and inhibited by addition of an OAT inhibitor to uremic plasma identifies IS as a major, poorly dialyzable, uremic toxin. The signaling pathways initiated by IS and possibly other solutes not effectively removed by dialysis may participate in the pathogenesis of renal scarring and uremic vasculopathy.

## Introduction

The dramatic improvement in uremic symptoms following hemodialysis treatment in patients with acute renal failure [1] and the demonstration that patients with chronic renal failure could be maintained by chronic hemodialysis [2], contributed greatly to the assumption that uremia was attributable to a small, water soluble substance or substances that could be removed by diffusion across a synthetic dialysis membrane. Urea and creatinine were seen as surrogate markers for filterable uremic toxins and hemodialysis was termed “renal replacement therapy”, (RRT). However, major features of chronic renal failure are largely unaffected by hemodialysis [3–6]. Patients undergoing hemodialysis or peritoneal dialysis have accelerated cardiovascular disease and progressive scarring of the diseased kidney with loss of residual renal function and, ultimately, anuria. Only 52 percent of dialysis patients are still alive three years after the start of treatment by hemodialysis or peritoneal dialysis, with deaths largely secondary to accelerated cardiovascular disease [6,7].

Varying the porosity of dialysis membranes, techniques of hemodialysis, dialysis time and dialysis frequency, while resulting in improved urea and creatinine removal, have resulted in only modest improvements in survival [8–10]. These observations have led to a reevaluation of the contribution of protein-bound or “middle molecules” not effectively removed by conventional dialysis [11]. The European Uremic Toxin (EUTox) Work Group cataloged 88 substances found at higher concentrations in the plasma of uremic patients than in normal individuals, including common solutes such as creatinine and urea [12]. Of these, 46% are free water-soluble low molecular weight compounds, 28% represent “middle” molecules too large to be dialyzed with “conventional techniques”, and 25% represent poorly-dialyzable protein-bound solutes [12–15]. Indoxyl sulfate, an aryl amine, has been identified as a potential uremic toxin responsible for accelerated renal scarring in the rodent remnant kidney model [16–19]. Elevated concentrations of IS have been found in patients with chronic renal failure [19]. It is highly bound to Sudlow site II of albumin which greatly limits filtration across the glomerular capillary membrane and diffusion across conventional synthetic dialysis membranes. The major mechanism responsible for renal excretion of protein-bound solutes is secretion by proximal renal tubular cells [20]. Marquez et al. reported the renal clearance of IS to average 40–51% of the clearance of urea in normal subjects despite 90% binding of IS, indicating renal tubular secretion as the major mechanism responsible for IS excretion [21].

To examine the efficiency of hemodialysis in the clearance of poorly filtered protein—bound uremic solutes, indoxyl sulfate and possibly others, we compared gene expression in cultured normal human renal cortical cells incubated in pre- and post-dialysis uremic plasma as a reporter system. Renal cortical cells were chosen as a reporter system because the renal tubule, known to be rich in OATs, is both the site of renal excretion of indoxyl sulfate and possibly a target for the toxic effects of IS [22,23].

## Materials and Methods

Heparinized plasma samples were obtained from 10 uremic patients undergoing thrice weekly hemodialysis (Table 1) and 5 control subjects without known renal disease. In uremic patients, blood was drawn from the afferent dialysis access line immediately pre- and post-dialysis.

**Table 1:** Characteristics of subjects with ESRD.

		Patient Characteristics					Plasma Markers				
	Patient	Gender	Age (Years)	Etiology	Time on Dialysis (Months	24 Hour Urine Output (Ounces)	Pre-D Cr^1^ (mg/ml)	Pre-D BUN (mg/dl)	Post-D BUN (mg/dl)	Pre-D IS^2^ (μg/ml)	Post-D IS (μg/ml)
Residual Renal Function	1	M	47	DM^3^	36	12–18	9.38	63	20	70.21	44.14
	2	M	70	DM	108	18–24	6.1	55	14	42.67	26.7
	3	F	64	DM	3	18	4.45	42	7	12.32	5.71
	4	M	59	DM	60	12	4.27	38	11	28.55	18.63
	5	M	42	DM	7	9	7.25	63	21	28.33	19.41
No Residual Renal Function	6	M	28	Transplant Rejection	36	0	13.28	47	12	79.56	40.2
	7	M	39	FSGS^4^	36	1	16.86	71	19	46.49	34.15
	8	M	53	DM	84	1	7.98	63	15	65.09	44.29
	9	F	32	DM	48	0	7.6	45	10	68.56	30.23
	10	M	50	HTN^5^	180	0	8.58	73	18	27.89	13.07

The preponderance of patients with diabetic glomerulosclerosis reflects the exclusion of patients with active glomerulonephritis or other inflammatory processes and the predominance of diabetic glomeruosclerosis in our dialysis population. Abbreviations: 1 = Creatinine, 2 = indoxyl sulfate, 3 = diabetes mellitus, 4 = focal segmental glomerulosclerosis, 5 = hypertensive nephropathy.

The effects of uremic plasma on gene expression were assessed in cultured human renal cortical cells. Plasma IS concentration was measured by MS-HPLC.

The study was approved by the New York University Institutional Review Board. Patients were recruited from the Lower Manhattan Dialysis Center in NYC. All participants provided witnessed, written informed consent to participate in this study. The consent forms, which were approved by the NYU IRB, were maintained in the files of the Principal Investigator.

### Cells and Incubation

Non-immortalized human renal cortical cells were purchased from Innovative BioTherapies (Ann Arbor, MI). These human cells, were isolated and cultured in the same manner as porcine cells, previously described [24], which were reported to retain transport properties typical for proximal tubular cells. Bicarbonate transport was decreased with acetazolamide, active glucose transport was suppressed with phlorizin, Na+/K ATPase activity was inhibited by ouabain, and para-aminohippurate (PAH) secretion was diminished with the transport inhibitor, probenecid. Porcine and human renal cortical cells, isolated by the technique employed by Innovative BioTherapies, were observed grow to confluence as a homogeneous layer [25]. While the possibility that the cell preparation we employed contained non-epithelial cells, mesangial or vascular smooth muscle cells, was not excluded by immunofluorescence staining, the homogeneous confluent layer demonstrated previously [25] suggests that epithelial cells made up the major component of the renal cortical cells. Further, since we were employing the cells as a general reporter system and did not intend to study the particular responses of renal cortical epithelial cells, a possible admixture of non-epithelial cells should not confound our findings. Nevertheless, recognizing that the cell culture may possibly contain non-epithelial cells, we refer to these cultured cells as “renal cortical cells” rather than “renal tubular cells”

Cells were maintained in a freeze medium consisting of UltraMDCK medium (12–749Q, Lonza, Basel, Switzerland) with 10% (v/v) dimethylsulfoxide (DMSO) (D-2650, Sigma, St. Louis, MO) and stored in liquid nitrogen prior to use. Cells were quickly thawed and suspended in protein-free UltraMDCK medium supplemented with 1 mL/L insulin, transferrin, ethanolamine, and selenium (ITES) (17–839Z, Lonza), 0.7 μg/L triiodothyronine (T3) (T-6397, Sigma), 50 μg/L epidermal growth factor (EGF) (236-EG, R&D Systems,Minneapolis, MN)), 30 μg/L retinoic acid (RA) (R-2625, Sigma), and 10 mL/L antibiotic–antimycotic solution (15250, Invitrogen, Carlsbad, CA). Cells were grown on Type IV collagen plates (354233 BD Sciences, Franklin Lakes, NJ). When plated cells were confluent, a 20% concentration of plasma was added as follows: normal control plasma (n = 5), control plasma spiked with 60 μg/ml IS (Sigma) (n = 5), pre-dialysis uremic plasma (n = 10), post-dialysis uremic plasma (n = 10), pre-dialysis uremic plasma with 1 mM probenecid (Sigma) (n = 5), and post-dialysis uremic plasma with 1 mM probenecid (n = 5). Incubations were carried out at 37° C for 24 hours before RNA was harvested.

### Microarray Analysis of mRNA Abundance

Employing the human renal cortical cells as a reporter system, total cellular RNA was isolated using the Qiagen RNeasy Mini Kit (Boston, MA) and checked for integrity, purity and quantity by UV spectroscopy and on the 2100 Agilent Bioanalyzer (Santa Clara, CA). The Affymetrix GeneChip WT Terminal Labeling and Controls Kit (Cleveland, OH), combined with the Ambion WT Expression Kit were used to prepare the labeled target population, following the manufacturer’s protocol. Biotin-labeled and fragmented cRNA hybridized to Affymetrix Genechip Human Gene 1.0 ST arrays and scanned using standard techniques recommended by the manufacturer. Feature intensity was extracted by GeneChip Operating System as CEL files. The probe-level analysis of the CEL files was done by the ExonRMA16 algorithm including quantile normalization using GeneSpring GX11 program. The raw array intensities were normalized using robust multichip average (RMA) using GeneSpring GX11 software (Agilent Technologies, Palo Alto, USA). The raw data is deposited in the NCBI GEO database under the identifier GSE45709.

### NanoString verification of gene array data

Twenty two genes were selected for comparison of gene expression by gene array and Nanostring analysis (Seattle, WA). Genes that were considered prominent in signaling pathways of interest and observed to exhibit varying degrees of dysregulation in gene arrays created following exposure to pre- and post-dialysis plasma were selected. The Nanostring method attaches a unique fluorescent “tag” to selected genes and quantitates the number of genes bearing the tag and is therefore an independent measure of gene expression [26]. The ratio of the log of the abundance (uremic/control, pre- and post-dialysis) for each of the 22 genes selected for comparison of Gene array and Nanostring analyses are displayed in Table 2.

**Table 2:** Comparison of gene microarray and Nanostring stimates of IS.

Gene Symbol	Pre-D/Ctrl Microarray	Post-D/Ctrl Microarray	Pre-D/Ctrl NanoString	Post-D/Ctrl NanoString	Gene Name
MKI67	2.03	2.26	1.62	1.8	marker of proliferation Ki-67
KIF11	1.69	1.8	1.37	1.48	kinesin family member 11
PLAT	1.55	1.78	1.31	1.5	tissue plasminogen activator
SERPINE1	1.44	1.68	1.67	2.3	plasminogen activator inhibitor
TNFSF13B	1.29	1.43	1.37	1.67	tumor necrosis factor (ligand) superfamily
BRCA2	1.28	1.48	1.3	1.58	breast cancer 2, early onset 1
IL8	1.27	1.31	1.37	1.46	IL8_human
CCL2	1.25	1.3	1.75	1.57	chemokine (C-C motif) ligand 2
THBS1	1.23	1.27	1.32	1.35	thrombospondin 1
FOSL1	1.2	1.35	1.37	1.69	FOS-like antigen 1
CA9	1.19	1.02	1.35	1.37	carbonic anhydrase IX
B4GALNT2	1.15	1.27	1.23	1.32	beta-1,4-N-aetyl-galactosaminyl transferase 2
ARHGDIB	1.14	1.17	1.12	−1.06	rho GDP dissociation inhibitor (GDI) beta
FGF1	1.14	1.26	1.24	1.31	fibroblast growth factor 1 (acidic)
TPM1	1.11	1.14	1.1	1.22	tropomyosin 1 (alpha)
RHOQ	−1.12	−1.16	−1.2	−1.23	ras homolog family member Q
PPARGC1A	−1.14	−1.19	−1.33	−1.45	peroxisome proliferator-activated receptor gamma
ME1	−1.14	−1.13	−1.23	−1.24	malic enzyme 1, NADP(+)-dependent, cytosolic 1
ACOX2	−1.19	−1.29	−1.42	−1.36	acyl-CoA oxidase 2, branched chain
GCLC	−1.24	−1.26	−1.48	−1.6	glutamate-cysteine ligase, catalytic subunit
GHR	−1.33	−1.36	−1.45	−1.33	growth hormone receptor
GPD1	−1.37	−1.64	−1.47	−1.88	glycerol-3-phosphate dehydrogenase 1

22 Genes are listed in descending order from greatest up-regulated to greatest down-regulated.The pre-and post dialysis values, used to create Fig. 2A and 2B are presented. Gene symbols are shown on the left; gene names are shown on the right.

### Measurement of Indoxyl Sulfate

High-performance liquid chromatography with an ABI Kratos 980 Fluorescence detector and Pyramid Axxiom software, was used to measure the concentration of IS in plasma samples. IS was dissolved in methanol to create a standard curve against which to test the plasma samples. Measurement of free IS concentration was performed on plasma ultrafiltrate (Centrifree Ultrafiltration, 10kDA Amicon filter), separated at 1000 rpm).

## Results

### Gene Expression Pre- and Post-Dialysis

The gene expression data was examined utilizing the Pavlidis Template Matching (PTM) method [27] to compare the gene expression in our cultured human renal cortical cells incubated with either control (n = 5), pre-dialysis plasma (n = 10), post-dialysis plasma (n = 10), control plasma spiked with IS (n = 5), pre-dialysis plasma with added probenecid (n = 5) and post-dialysis plasma with added probenecid (n = 5).

**Fig 1:**
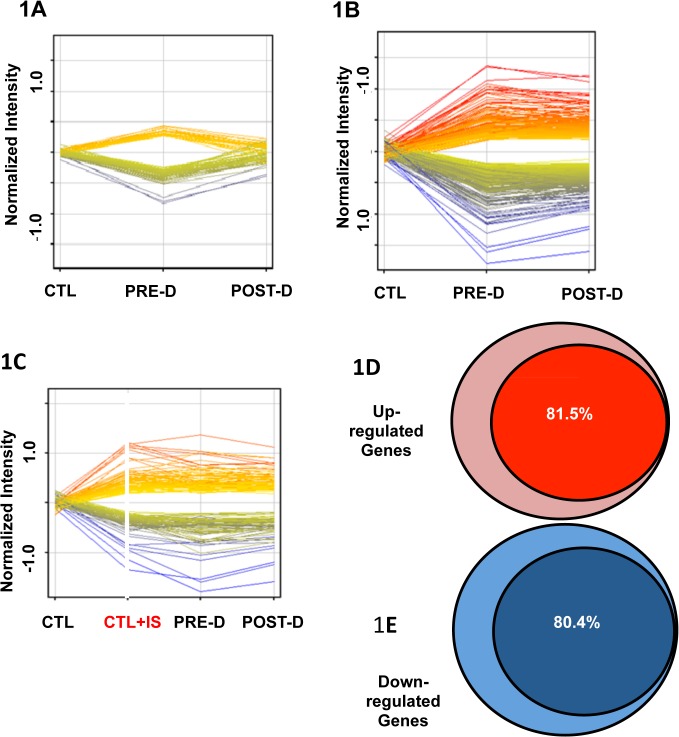
Gene expression in reporter renal tubular cells. A: Genes that returned to baseline post-dialysis. Each individual line represents the average value in the 10 uremic subjects for each gene that was dysregulated. 282 genes were upregulated (displayed as yellow to red lines) and 255 genes were downregulated (displayed as green to blue lines) in the pre-dialysis samples as compared to normal controls. Post-dialysis these values returned to baseline. B: Genes that remained dysregulated after dialysis treatment. 843 genes were upregulated and 532 genes were downregulated. C: Genes expressed following incubation in normal plasma spiked with indoxyl sulfate compared with their expression levels in cells treated with pre-dialysis and post-dialysis plasma. 908 genes were upregulated and 571 genes were downregulated. D: 81.5% of upregulated genes that were not normalized by dialysis were mimicked by addition of indoxyl sulfate to normal plasma. E: 80.4% of downregulated genes that were not normalized by dialysis were mimicked by addition of indoxyl sulfate to normal plasma.

After creating the (PTM) templates, we *arbitrarily* set 10% as the boundary defining *dysregulation* (up-regulation or down-regulation). Fig. 1A and 1B demonstrate the expression of 1912 genes that exhibited at least a 10% difference, either increased or decreased expression as compared with normal controls, when reporter renal cortical cells were incubated with pre- and post-dialysis uremic plasma. The expression of 537 dysregulated genes returned to baseline when cells were incubated with post-dialysis plasma (Fig. 1A). Many more genes (1375) remained dysregulated when incubated with post-dialysis plasma (Fig. 1B). Using the more rigorous criterion for altered gene expression of at least 20% deviation from control, the findings were not significantly different; 1479 genes exhibited dysregulation that was not corrected by a single dialysis (Fig. 1C). This gene dysregulation, not corrected by dialysis, strongly suggests the presence of one or more poorly-dialyzable solutes affecting gene expression.

To examine the role of indoxyl sulfate on the altered gene expression induced by incubation in uremic plasma in our renal cell reporter system, IS was added to control plasma to a concentration of 60 μg/ml (Fig. 1C). Strikingly, 81.5% of the genes that that remained up-regulated (Fig. 1D) and 80.4% of the genes that remained down-regulated with incubation in post-dialysis uremic plasma (Fig. 1E) were mimicked by the addition of IS to control plasma. This finding identifies indoxyl sulfate as a major candidate uremic toxin.

### Comparison of microarray and NanoString quantification

**Fig 2:**
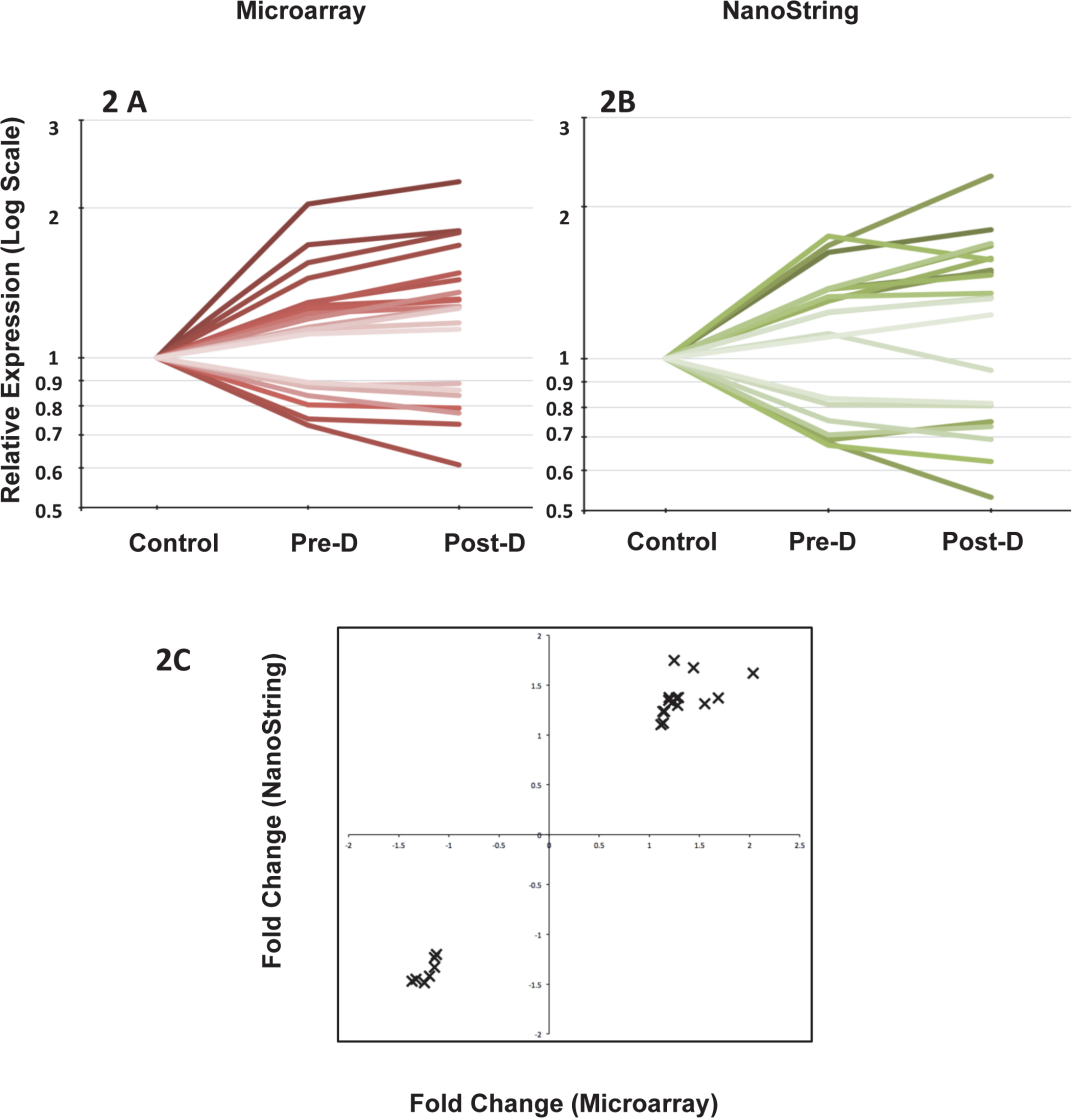
Comparison of microrray findings with Nanostring assay (22 genes). 2A: Changes in gene expression estimated by microarray. 2B: Changes in gene expression estimated by Nanostring. 2C: Comparison between microarray estimate and Nanostring shown on a coordinate plot. Pearson correlation coefficient, pre-D/Control = 0.98, post-D/Control = 0.92.

There was a strong concordance between gene expression estimated from gene arrays and measured by quantitative verification experiments using NanoString (Fig. 2) in all 22 genes over a wide range of over- or under- expression. (Table 2, Fig. 2, Pearson correlation coefficient pre-dial/control = 0.98, post dial/control = 0.92).

### Effects of OAT inhibition on the effects of indoxyl sulfate

**Fig 3:**
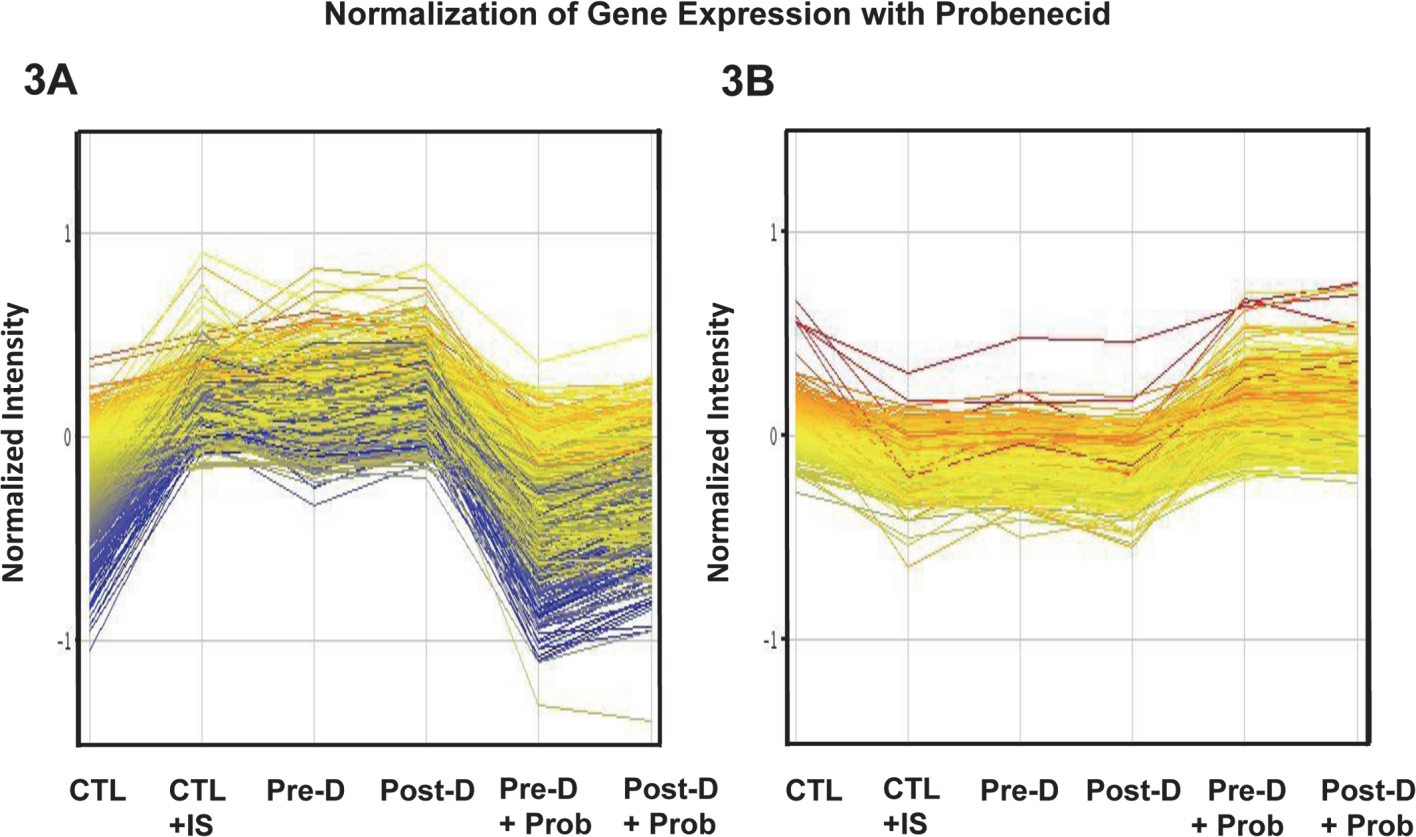
Effects of probenecid on gene expression. A: Genes up-regulated in cells incubated in pre- and post dialysis plasma returned toward baseline with added probenecid. B: Genes down-regulated in cells incubated in pre- and post dialysis plasma returned toward baseline with added probenecid. Each line represents the average of 5 or 10 gene arrays.

When probenecid, an inhibitor of OATs [22], was added to pre- and post-dialysis uremic plasma, it abolished the gene dysregulation observed when cells were incubated with uremic plasma (Fig. 3). Although the effects of probenecid are not specific for OAT 1 or OAT 3, this finding lends support to the hypothesis that the effects of indoxyl sulfate are medicated through OAT transport.

### Indoxyl Sulfate Concentration

**Fig 4:**
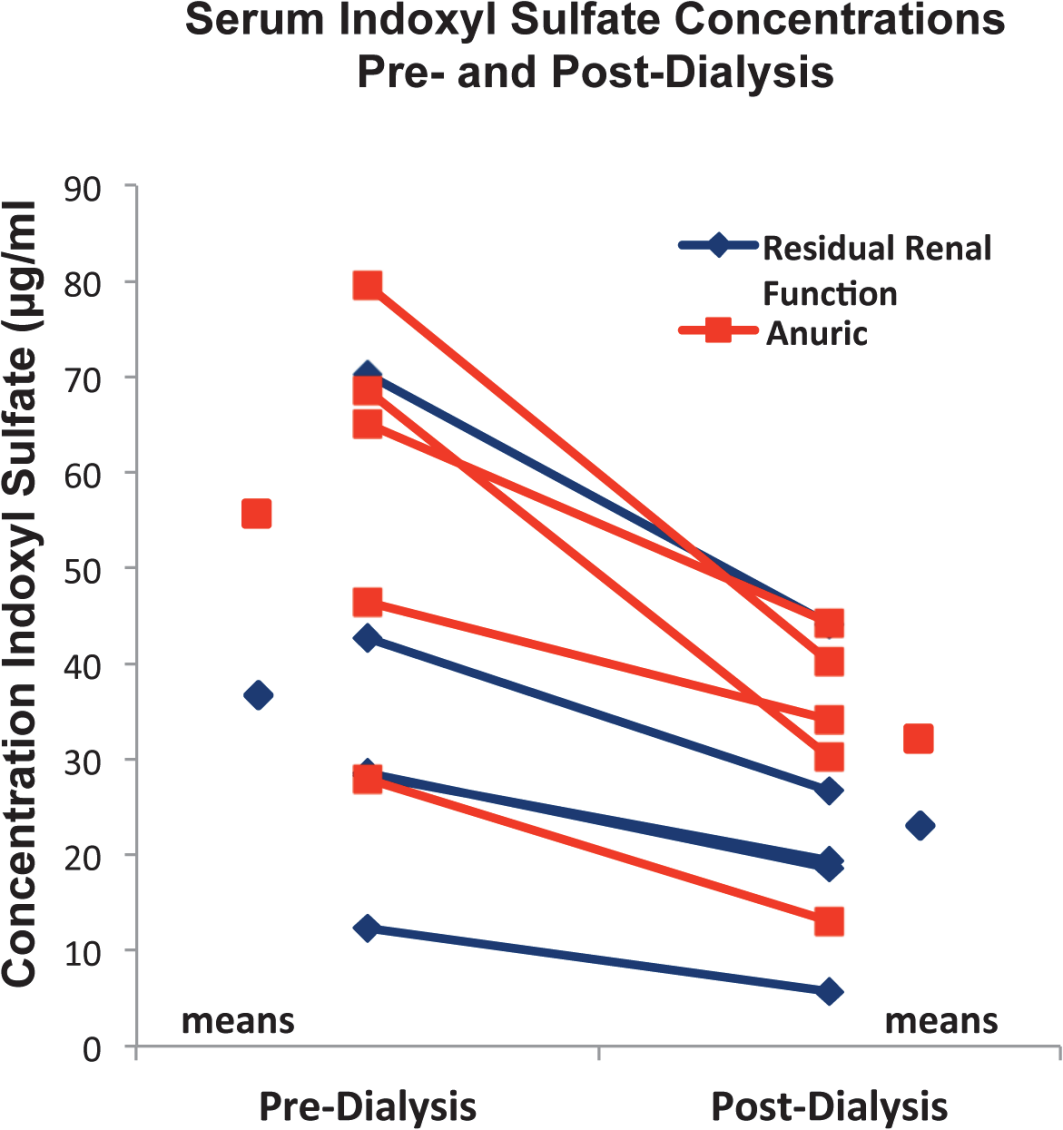
Plasma indoxyl sulfate concentration pre- and post—hemodialysis. Patients with and without residual renal function exhibited a decrease in circulating total indoxyl sulfate levels post-dialysis. Total indoxyl sulfate concentration was greater in both pre- and post-dialysis plasma in subjects without Residual Renal Function (anuric) than in subjects with Residual Renal Function.

Both pre- and post-dialysis plasma IS concentrations were markedly elevated compared to control (0.89 μg/ml, p<0.001) (Table 1, Fig. 4). The mean total IS concentration was 47.0 μg/ml in pre-dialysis plasma and 27.7 μg/ml in post-dialysis plasma. The difference (pre-vs post-dialysis) was significant (p < 0.05). The mean concentration of free (unbound) IS in pre-dialysis plasma was 13.5 μg/ml representing 26.8% of total IS. Reduced binding of indoxyl sulfate in uremic plasma has been reported by others [28,29]. While protein binding renders IS poorly dialyzable, the increased concentration of unbound IS in equilibrium with the markedly elevated concentration of protein-bound IS in uremic plasma and reduced binding affinity in uremic plasma [28] facilitate the removal of IS by filtration during dialysis and could fully account for the observed decrease in total indoxyl sulfate in post-dialysis plasma.

### Residual Renal Function

**Fig 5:**
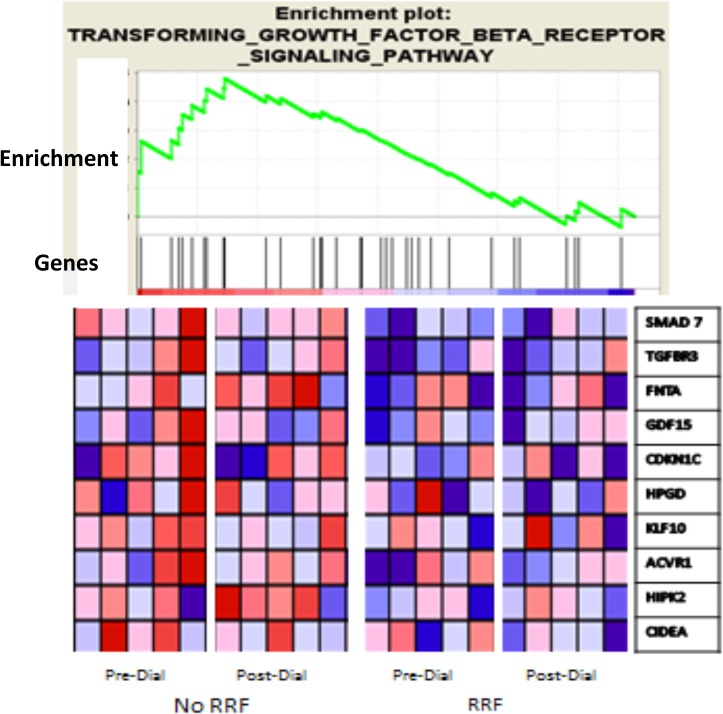
The expression of genes in the TGF receptor pathway identified by Pavlidis Template Matching [27] in the DAVID data base [31] and subjected to Gene Set Enrichment Analysis (GSEA) [32,33]. The 10 uremic patients were dichotomized into those without RRF (n = 5) on the left and those with RF (n = 5) on the right. The GSEA enrichment score curve (top panel) for TGF-beta receptor signaling (34 genes), indicates 10 genes as core-enriched (mapped on ranked list left of the peak on the enrichment score curve). The activation of these 10 genes was greater following incubation in pre- and post- dialysis (Pre-Dial, Post-Dial) plasma of subjects without RRF than in subjects with RRF (bottom panels, red in heat map = high mRNA abundance, blue = low abundance).

In this series 5 subjects were judged to have residual renal function (RRF),based on self-reported daily urine output of 2 or more ounces and 5 subjects were anuric (Table 1). Patients with RRF had lower pre- (36.4 μg/ml) and post-dialysis (22.9 μg/ml) plasma concentrations of IS (total) than patients without RRF (pre-dialysis 55.9 μg/ml, post-dialysis 32.4 μg/ml) (Table 1, Fig. 4). While these differences between subjects with and without RRF did not reach statistical significance in this small sample, the gene array data shed some light on the importance of RRF in patients undergoing chronic dialysis. The expression of genes prominent in one TGF beta signaling pathway, which has long been thought to be involved in progressive renal disease [30], demonstrated greater activation by pre- and post- dialysis plasma of patients lacking RRF than plasma of patients with RRF (Fig. 5).

## Discussion

Our finding that almost 2000 genes expressed in human renal cortical cells are dysregulated when exposed to uremic plasma is in agreement with the earlier studies of Aoyama, Enomoto, and Niwa, who reported gene dysregulation in renal cortex of 5/6 nephrectomized uremic rats [34].

The most important conclusions of the present study follow from the observation that there are two classes of substances in the plasma of patients with uremia that effect gene expression in normal human renal cortical cells. One class of molecules appears to be effectively removed by conventional hemodialysis. A second class of uremic solutes, not effectively removed during hemodialysis, accounted for approximately three quarters of the altered gene expression observed in our human renal cortical cell reporter system. This strongly suggests the effects of one or many poorly-dialyzable uremic solutes, of which IS has been considered a candidate [11–17]. Elevated plasma indoxyl sulfate concentration in uremia is a well-established fact [12,14,17] and it is known that indoxyl sulfate is poorly-dialyzable [11–13,21].The present finding that the protein-bound aryl amine, IS, added to control plasma mimicked more than 80% of the dialysis-resistant altered gene expression, constitutes additional strong evidence of a unique and important role for IS as a uremic toxin. Our finding that probenecid blocked the effects of uremia on gene dysregulation, identifies OATs as important for the membrane transport of this uremic toxin.

The role of IS as a *major* uremic solute is supported by the study of Rhee et al [35]. Employing a metabolomics approach, they identified 38 putative polar uremic solutes (i.e., candidate uremic toxins) elevated in the predialysis plasma of patients with ESRD. All but 2 were cleared by a single hemodialysis treatment with an efficiency roughly comparable to the clearance of creatinine, i.e., they were cleared by diffusion across the dialysis membrane. Only IS and choline, fit the definition of a “poorly dialyzable protein-bound” uremic solute; plasma IS concentration declined by only 17% following dialysis in Rhee’s study.

Our finding that a great number of genes are dysregulated by indoxyyl sulfate is consistent with other evidence that IS activates one or more transcription factors. Studies of the “downstream” effects of IS [36], the mechanism of gene activation or inactivation and the consequences of gene dysregulation have largely been carried out in cultured cells [11]. IS acts by binding to the cytoplasmic aryl hydrocarbon receptor (AHR) [36,37]. The complex translocates to the nucleus, where it forms a heterodimer with aryl hydrocarbon receptor nuclear translocator (ARNT), a potent transcription factor [37]. Gonduin et al., studying the effects of IS on human umbilical vein endothelial cells, reported that blocking the action of AHR by inhibiting its chaperone protein or silencing AHR expression with small interfering RNA (siRNA) significantly reduced the downstream effects of IS on the endothelial target cells [36].

Finally, we report here that plasma IS concentration is greater in anuric patients than in patients who retain RRF. A similar difference in plasma IS concentration among patients with and without RRF has been reported by Klammt et al. [28] and Marquez et al. [21]. Analysis of large data sets suggest that patients with RRF, i.e., continued urine production while undergoing hemodialysis or peritoneal dialysis, have better survival and less cardiovascular disease than patients who are anuric [38,39]. It has been estimated that RRF provides clearance of the conventional surrogate uremic markers, urea and creatinine, roughly equivalent to prolonging each hemodialysis by only 33 minutes [39]. The findings of less severe cardiovascular disease and the observation that survival is virtually independent of the dialyzer clearance of creatinine in patients with RRF [39] suggest the possibility that RRF confers a cardiovascular benefit to patients with ESRD and suggests a unique mechanism for continued urine production when glomerular filtration is markedly reduced.

Grantham described the secretion of fluid by *isolated rabbit renal tubules* and found that the rate of this “*urine production*” was increased when either para-amino hippurate (PAH) or uremic plasma was added to the bathing medium [40]. He speculated that “under conditions of markedly reduced…glomerular filtration, mammalian proximal tubules could secrete…some of the potentially toxic products normally excreted by the kidney…” which would “serve a useful survival function” [40]. It does not seem far-fetched to speculate that RRF may represent the tubular secretion of IS and osmotically obligated fluid in the proximal renal tubule rather than urine generated by glomerular filtration in residual intact functioning nephrons

Although the studies reported here represent data obtained in only 10 uremic subjects and 5 controls, we believe the findings are sufficiently rigorous and in accord with published findings describing the dialysis of IS and elevated IS concentrations in patients with advanced renal failure, and with observations in the rodent remnant kidney model, to lead us to suggest that indoxyl sulfate is the major poorly-dialyzed uremic solute. As such, these findings dictate a significant paradigm shift in our understanding of the role of protein-bound, poorly dialyzable, uremic solutes in the pathogenesis of vascular disease and renal scarring in chronic renal failure. Our findings open the way to the investigation of new treatments that are radically different from those, focused on the dialytic removal of uremic toxins, which have dominated the field of nephrology for the past 60 years. These might include measures aimed to alter the expression of OAT transporters [41], decrease production of indole by perturbing the gut microbiome with antibiotics or probiotics [42], decrease intestinal absorption of indole [43], or interfere with the protein binding of IS [44]. Further, they suggest a new role for RRF. If RRF contributes significantly to the elimination of IS and related uremic toxins, it would dictate that attention be paid to the avoidance of drugs that might compete for transport by the OAT transporters [45].
